# Einflüsse von Klimawandel und Wetter auf die körperliche Aktivität

**DOI:** 10.1007/s00103-026-04314-7

**Published:** 2026-10-15

**Authors:** Sven Schneider, Selina Mencocco

**Affiliations:** Zentrum für Präventivmedizin und Digitale Gesundheit (CPD), Alte Brauerei, Medizinische Fakultät Mannheim der Universität Heidelberg, Röntgenstraße 7, 68167 Mannheim, Deutschland

**Keywords:** Hitze, Hohe Temperaturen, Klimawandel, Körperliche Aktivität, Bewegung, Heat, High temperatures, Climate change, Physical activity, Exercise

## Abstract

Schon heute wird aus Public-Health-Sicht das gesundheitsförderliche Potenzial körperlicher Bewegung nicht ausreichend genutzt. Der Temperaturanstieg und weitere klimatische Veränderungen könnten bestehende Bewegungsdefizite zusätzlich verstärken. Die vorliegende narrative Übersicht untersucht daher, welche Relevanz Klima und Wetter für die körperliche Aktivität haben und wie sich der Klimawandel künftig auf das Bewegungsverhalten auswirken könnte.

Zahlreiche retrospektive Studien belegen insbesondere die Bedeutung der Lufttemperatur. Während steigende Außentemperaturen Bewegung bis zu einem gewissen Punkt begünstigen, nimmt sie bei hohen Temperaturen wieder ab. Studien zeigen zudem, dass körperliche Aktivität während akuter Hitzewellen deutlich eingeschränkt sein kann. Der typische umgekehrt u‑förmige Zusammenhang zwischen Lufttemperatur und Bewegung erreicht seinen Scheitelpunkt bei etwa 26 °C bis 29 °C. Er wird jedoch durch weitere Klimaelemente sowie individuelle, zeitliche und räumliche Faktoren beeinflusst, was Prognosen des künftigen Bewegungsverhaltens erschwert. Während sich körperliche Aktivität retrospektiv gut modellieren lässt, kommen Projektionen daher zu unterschiedlichen, teils widersprüchlichen Ergebnissen.

Bislang bleibt offen, in welchen Zeiträumen und Settings der fortschreitende Klimawandel insgesamt zu einer Zu- oder einer Abnahme körperlicher Aktivität führen wird, etwa in Deutschland. Unabhängig davon sollten Präventionsansätze wie Hitzeaktionspläne und Musterhitzeschutzpläne darauf abzielen, einer weiteren Verstärkung bestehender Bewegungsdefizite insbesondere bei vulnerablen Gruppen im Sommer und bei Hitzewellen entgegenzuwirken.

## Abstract (en)

From a public health perspective, the health-promoting potential of physical activity is already not being fully utilized. Rising temperatures and other climate changes could further exacerbate existing physical inactivity. This narrative review therefore examines the relevance of climate and weather to physical activity and how climate change could affect physical activity patterns in the future.

Numerous retrospective studies demonstrate, in particular, the importance of air temperature. While rising outdoor temperatures promote physical activity up to a certain point, activity levels decline again at high temperatures. Studies also show that physical activity during acute heat waves can be significantly reduced. The typical inverted U‑shaped relationship between air temperature and physical activity peaks at approximately 26 °C to 29 °C. However, it is influenced by other climatic elements as well as individual, temporal, and spatial factors, which complicates forecasts of future physical activity patterns. While physical activity can be modeled well retrospectively, projections therefore yield varying and sometimes contradictory results.

It remains unclear at this point over what time periods and in what settings ongoing climate change will lead, overall, to an increase or a decrease in physical activity—for example, in Germany. Regardless, prevention strategies such as heat action plans and model heat protection plans should aim to counteract a further exacerbation of existing physical inactivity, particularly among vulnerable groups during the summer and heat waves.

## Hintergrund

Ohne Bewegung keine Gesundheit: Regelmäßige körperliche Aktivität trägt in jedem Alter zur Gesundheit bei. So ist ihr protektiver Effekt auf die Entstehung von Erkrankungen wie Bluthochdruck, Herzinfarkt, Schlaganfall, das metabolische Syndrom und Diabetes mellitus ebenso wie auf degenerative Erkrankungen bis hin zu Krebserkrankungen unbestritten. Darüber hinaus hat körperliche Aktivität positive Effekte auf wichtige kognitive, emotionale und motivationale Kompetenzen [1, 2]. Nicht zuletzt ist körperliche Aktivität eine Voraussetzung für die Mobilität des Menschen und somit auch für seine Teilhabe am gesellschaftlichen Leben [2].

Initiativen zur Bewegungsförderung wie die Nationalen Empfehlungen für Bewegung und Bewegungsförderung (NEBB; [2]) und der „Runde Tisch Bewegung und Gesundheit“ unter Leitung des Bundesgesundheitsministeriums [3] zielen auf diese vielfältigen und sich verstärkenden Wirkungen ab. Dabei verfolgt die Bundesregierung einen sektorübergreifenden Ansatz, der Bewegung im Alltag ebenso wie sportliche Aktivität umfasst.

Der globale Klimawandel könnte das gesundheitsfördernde Potenzial körperlicher Aktivität gefährden [4, 5]. Neben seinen massiven und komplexen Auswirkungen auf Ökosysteme, Gesellschaft und Wirtschaft werden in den letzten Jahren zunehmend auch seine klimaspezifischen Auswirkungen auf die Gesundheit erkannt. Laut Weltgesundheitsorganisation zählen etwa Sportler*innen ebenso wie Außenbeschäftigte, die einer körperlichen Tätigkeit nachgehen, zu den bedeutendsten Risikogruppen hinsichtlich der gesundheitlichen Folgen des Klimawandels [4].

Vor diesem Hintergrund befasst sich die vorliegende narrative Übersicht mit der Frage, welche Auswirkungen der fortschreitende Klimawandel auf das Bewegungsverhalten der Zukunft haben könnte. Dazu werden zunächst zentrale Begriffe definiert, Bewegungsdefizite der deutschen Bevölkerung dargestellt und mögliche gesundheitliche Auswirkungen des Klimawandels zusammengefasst. Anschließend wird die internationale und nationale Befundlage zu der oben genannten Fragestellung erörtert. Der Beitrag schließt mit einem konzeptionellen Modell, welches den Einfluss von Klima und Wetter auf das Ausmaß an körperlicher Aktivität innerhalb einzelner Bewegungsdomänen synoptisch darstellt.

Bei der Literatursuche wurden die Datenbanken Medline, PubMed sowie das Sportinformationsportal des Bundesinstituts für Sportwissenschaft (BISp) „SURF“ verwendet, welches die Datenbanken SPOLIT, SPOFOR, SPOMEDIA und den Fachinfoführer Sport umfasst. Die Suche erfolgte zwischen dem 8. und dem 16.04.2026 in Titel und Abstract (TIAB) mittels der Schlüsselwörter „Sport“, „Bewegung*“ und „Klimawandel“. Zudem wurden die Literaturlisten der Treffer ausgewertet.

## Begriffsbestimmung von „Bewegung“

Den Nationalen Empfehlungen für Bewegung und Bewegungsförderung entsprechend werden die Begriffe „Bewegung“ und „körperliche Aktivität“ im Folgenden synonym verwendet. Bewegung bzw. körperliche Aktivität wird demnach als eine durch die Skelettmuskulatur erzeugte Bewegung des menschlichen Körpers verstanden, die zu einem substanziellen Anstieg des Energieverbrauchs über den Ruheenergieverbrauch (also über ein metabolisches Äquivalent) hinaus führt [2].

Aus Public-Health-Perspektive umfasst dieser Bewegungsbegriff mehrere sogenannte Bewegungsdomänen. Eine dieser Domänen ist die körperliche Aktivität in der Freizeit. Dies schließt Sport in all seinen Organisationsformen ein. Dazu zählen der organisierte Sport als Schulsport, kommerzieller Sport und Vereinssport ebenso wie nicht organisiertes, individuelles Sporttreiben, etwa beim Joggen, Skifahren, Inlineskaten und Bouldern. Eine weitere Domäne ist die körperliche Aktivität zur Fortbewegung und zum Transport, also beispielsweise das Gehen oder Radfahren, um Strecken zur Schule, zur Arbeit oder zum Einkaufen zu überwinden. Weitere Domänen sind die körperliche Aktivität im Haushalt (z. B. beim Aufräumen oder bei der Gartenarbeit) sowie bei der Erwerbsarbeit (z. B. als Gärtner*in, Postzusteller*in, Straßenbauer*in oder Pflegekraft; [3]).

## Aktuelle Bewegungsdefizite

Die Empfehlungen der Weltgesundheitsorganisation zu Ausdaueraktivitäten werden nach bevölkerungsweiten Repräsentativdaten des Robert Koch-Instituts von 26 % der Kinder und Jugendlichen zwischen 3 und 17 Jahren, von 52 % der Erwachsenen zwischen 18 und 64 Jahren und von 39 % der Erwachsenen ab 65 Jahren erfüllt [6]. Demnach bewegt sich aktuell ein großer Teil der bundesdeutschen Bevölkerung zu wenig. Die Autor*innen jener aktuellen Übersichtsarbeit betonen, dass Mädchen und Frauen ebenso wie Personen mit höherem Alter, Migrationshintergrund, niedrigerem Einkommen oder geringerem Bildungsniveau die deutlichsten Bewegungsdefizite aufweisen [6]. Aufgrund unterschiedlicher Studiendesigns und Messmethoden einerseits und spezifischer Pandemieeffekte andererseits seien für Deutschland allerdings kaum valide Aussagen zu Veränderungen über die letzten Jahre und Jahrzehnte möglich. International scheint der Anteil inaktiver Erwachsener über die letzten Dekaden allerdings deutlich angestiegen zu sein [6].

## Gesundheitliche Auswirkungen des Klimawandels

Der Klimawandel manifestiert sich in der Veränderung der langfristigen Klima- und der kurzfristigen Wetterelemente [7]. Die wichtigsten Elemente sind Temperatur, Niederschlag, Wind, Luftfeuchte, Bewölkung, Luftdruck, Sonnenscheindauer respektive Wärme- und UV-Strahlung [8]. So nahm in Deutschland die Zahl heißer Tage seit 1951 um 208 % zu, während die Zahl an Eistagen im gleichen Zeitraum um 57 % und die Zahl an Schneetagen um 53 % zurückgegangen ist. Im gleichen Zeitraum nahmen Starkregenereignisse um 6 % und die Sonnenscheindauer um 14 % zu [9].

Diese klimatischen Veränderungen manifestieren sich in aktuellen Wetterlagen und können unterschiedliche Auswirkungen auf die Gesundheit haben: Als direkte gesundheitliche Folgen des Klimawandels werden eine Zunahme an Hitzeerkrankungen und Hitzenotfällen, mehr Unfallrisiken durch Extremwetterereignisse sowie eine Zunahme an UV-bedingten Hautkrebserkrankungen beschrieben [10]. Indirekte gesundheitliche Folgen des Klimawandels werden über Veränderungen in Ökosystemen vermittelt. So werden im Zuge des Klimawandels inhalative Risiken (etwa durch Ozon und Pollen) potenziell steigen. Durch den Klimawandel verursachte Dürren begünstigen Vegetationsbrände, welche die Feinstaubbelastung auch großflächig erhöhen können. Eine mildere Witterung und die Ausbreitung gebietsfremder Arten belasten die Luft durch Allergene. Hinzu kommen vermehrte Infektionsrisiken z. B. durch Vektoren wie Zecken und Stechmücken sowie durch wasserbürtige oder lebensmittelassoziierte Infektionen [10]. Nicht zuletzt hat der Klimawandel auch akute und chronische Folgen für die psychische Gesundheit [10]. Direkte Effekte (z. B. die Wirkung der Lufttemperatur) und indirekte Effekte (z. B. die Wirkung der Feinstaubbelastung oder die Sportmotivation) haben das Potenzial, die körperliche Aktivität auf Individual- und Populationsebene zu beeinflussen [11].

Gleichzeitig bedeutet die langfristige Änderung der Jahresdurchschnittstemperatur hierzulande nicht nur längere, häufigere und intensivere Hitzewellen, sondern auch mildere Temperaturverläufe. So lag der Temperaturanstieg seit 1881 für den deutschen Frühling bei 2,1 °C und für den deutschen Winter bei 3,0 °C [5]. Die oft als „Behaglichkeitszunahme“ bezeichnete mildere Witterung begünstigt verschiedene Formen körperlicher Aktivität im Freien und ist deswegen bei der Betrachtung künftiger Bewegungstrends ebenfalls zu berücksichtigen [12].

Noch vor wenigen Jahren wurde der Einfluss des Klimawandels und einzelner Klimaelemente auf die körperliche Aktivität selbst in Publikationen der Weltgesundheitsorganisation in einschlägigen Modellen nicht berücksichtigt (so auch in einem Beitrag im *Bundesgesundheitsblatt*: [13]). In dem Maße, wie sich der Klimawandel beschleunigt, werden mittlerweile Forderungen lauter, nunmehr auch seine Auswirkungen auf Quantität und Qualität körperlicher Aktivität wissenschaftlich zu ergründen [1, 7, 14–16].

## Klima, Wetter und körperliche Aktivität

### Lufttemperatur und körperliche Aktivität

Mit Abstand am häufigsten betrachtet wurde bis dato der Einfluss der Lufttemperatur auf körperliche Aktivität. Einschlägige Studien berücksichtigen dabei sowohl die Tatsache, dass Hitzewellen künftig häufiger, länger und intensiver auftreten werden, als auch die Veränderungen der jahreszeitlichen Durchschnittstemperaturen [11, 15, 17]. Nahezu alle von uns gesichteten retrospektiven Studien kommen zu demselben prägnanten Befund: Zwischen der Lufttemperatur und der körperlichen Aktivität betroffener Individuen besteht ein umgekehrt u‑förmiger Zusammenhang [18]. Während also körperliche Aktivität bis zu einem gewissen Punkt durch zunehmende Außentemperaturen begünstigt wird, kippt dieser Effekt ab einer gewissen Temperatur ins Gegenteil [11, 19].

So ist der besagte umgekehrt u‑förmige Verlauf der zentrale Befund aus einem Scoping-Review von über 100 Studien, die auf der Messung von Beschleunigung (Akzelerometern) oder von Schritten (Pedometern) basieren [20]. Lediglich die konkrete Temperatur, bei der sich der Scheitelpunkt der Verlaufskurve befindet, unterscheidet sich in den dort inkludierten Studien. Ein anderer Scoping-Review umfasste Studien unter Verwendung von Wearables (z. B. am Körper getragene Fitnesstracker), die neben körperlicher Aktivität auch Parameter wie die Außentemperatur erfassten. In der Gesamtschau wird dieser Peak, ab dem sich ein Rückgang körperlicher Aktivität abzeichnet, auf etwa 26 °C beziffert [21].

Zu einem ähnlichen Ergebnis kommt eine umfangreiche Studie aus den USA auf Basis von Befragungsdaten von 1,9 Mio. Personen: Demnach steige das Ausmaß körperlicher Aktivität bis zu einem Temperaturbereich um die 28–29 °C zunächst an, um dann anschließend geringfügig und ab 36 °C deutlich zurückzugehen [7]. In China lag der Temperaturpeak für den besagten Zusammenhang in 4 großen Städten zuzüglich Hongkong nach Auswertung digitaler Schrittzähler im Temperaturbereich zwischen 16 °C und 24 °C. Die Autor*innen erklären die vergleichsweise niedrigeren Scheitelpunkte unter anderem mit den an den Studienorten vorherrschenden feuchten subtropischen Klimata [16].

Die wenigen bis dato verfügbaren Daten aus Deutschland spiegeln die internationale Befundlage wider: So zeigen aktuelle Akzelerometriedaten der ActiFE (Activity and Function in Older People in Ulm)-Study, dass die dort untersuchten Über-65-Jährigen ihre alltägliche Bewegung ab einer Außentemperatur von etwa 25 °C reduzierten [1, 21]. Der Wert von 25 °C findet sich auch in Analysen der KORA-FIT-Studie in Augsburg: Die durchschnittlich 64 Jahre alten Studienteilnehmer*innen schränkten ihre körperliche Mobilität beim Überschreiten der 25 °C-Marke (und im Übrigen auch beim Unterschreiten der 7 °C-Marke) tendenziell ein [22].

### Weitere Wetterelemente und körperliche Aktivität

Einige Studien berücksichtigen in ihren Analysen auch weitere Wetterelemente. Gut belegt ist ein positiver Zusammenhang zwischen körperlicher Aktivität und der Sonnenscheindauer sowie ein mehrheitlich negativer Zusammenhang mit der Niederschlagsmenge, der Windgeschwindigkeit und der Luftfeuchtigkeit [14, 20]. Uneindeutige Befunde hingegen zeigen sich bezüglich der Elemente Schneefall, Bewölkung und Luftdruck [14, 20]. Auch in der oben erwähnten ActiFE-Study standen die Zahl der Sonnenstunden und die Strahlungsstärke in nahezu linearem positiven Zusammenhang mit der täglichen Bewegung, während mehr Regen, stärkerer Wind und höhere Luftfeuchtigkeit einen nahezu linearen negativen Einfluss auf das Bewegungsausmaß hatten [1, 21].

### Luftqualität und körperliche Aktivität

Klima und Wetter beeinflussen auch die Luftqualität. Anders als in anderen Regionen dieser Welt findet der Zusammenhang zwischen Luftverschmutzung und Bewegung in Deutschland bisher kaum Beachtung. Unter anderem in Australien, Amerika und Asien spielt die Feinstaubbelastung für das Ausmaß körperlicher Aktivität eine wichtige Rolle [11, 23]. So kommt ein systematischer Review von empirischen Studien aus China zu dem Schluss, dass neben der allgemeinen Luftqualität vor allem die Feinstaubbelastung (PM_2.5_) die körperliche Aktivität in verschiedenen Bewegungsdomänen negativ beeinflusst [23].

In Asien ebenso wie in Westaustralien und Nordamerika rechnet man in diesem Zusammenhang künftig mit einer deutlichen Zunahme der Feinstaubbelastung (PM_2.5_; [11]). Neben Dürreperioden und ausbleibenden Regenfällen, welche die Feinstaubbelastung insbesondere in den dortigen Großstädten durch Reifenabrieb, Dieselruß, Kamin- und Industrieabgase verstärken, könnten dort vor allem auch vermehrte großflächige Vegetationsbrände (etwa Wald- und Buschfeuer) zu einem weiteren Anstieg beitragen [11, 24]. So könnten Luftbelastung und Geruchsbelästigung per se ebenso wie amtliche Warnungen vor Vegetationsbränden respektive der Feinstaubbelastung die Motivation für Bewegung im Freien reduzieren.

In Deutschland und Europa gehen die meisten Projektionsstudien dagegen eher von einem lediglich geringfügigen Anstieg der PM_2,5_-Feinstaubkonzentration und von einer Abnahme der Konzentration von inhalierbarem Schwebstaub (PM_10_) aus. Zurückgeführt wird dies auf die bei uns im Zuge des Klimawandels zunehmenden Niederschläge im Winter, die insgesamt zunehmende Luftfeuchtigkeit sowie die Veränderung der Windmuster [24].

### Klimawandel und körperliche Aktivität

Die Frage nach dem grundsätzlichen Zusammenhang zwischen Wetterelementen, Luftschadstoffen und körperlicher Aktivität lässt sich auf Basis retrospektiver Analysen beantworten. In den obigen Abschnitten werden dazu zahlreiche Reviews und Einzelstudien aufgeführt. Die Frage nach der Bedeutung des Klimawandels für unser künftiges Bewegungsverhalten bedarf dagegen prognostischer Methoden. Einen solchen Ansatz verfolgt eine gerade erschienene Studie unter Nutzung von Paneldaten von 156 Nationen (darunter auch Deutschland) aus allen Kontinenten.

Unter Annahme des gemäß Weltklimarat ungünstigsten Szenarios (sog. „SSP5‑8.5 Szenario“) und unter Adjustierung weiterer Klimaelemente sei bis zum Jahr 2050 mit einer signifikanten Zunahme globaler körperlicher Inaktivität um 1,75 Prozentpunkte zu rechnen. Besonders betroffen von einem Rückgang körperlicher Aktivität im Zuge dieses ungünstigen Temperaturszenarios seien tropische und subtropische Regionen in Zentralamerika, der Karibik, der östlichen Subsahara und in Südostasien [15].

Eine weitere Prognosestudie kommt auch für Australien zu dem Schluss, dass sich die körperliche Aktivität insgesamt deutlich reduzieren wird: Bis zum Jahr 2070 wird dort ein Anstieg der Jahresdurchschnittstemperatur um 6 °C prognostiziert. Gemäß Modellrechnung würde eine dort lebende Person statt an bisher einem Tag in 5 Jahren dann an 9 Tagen pro Jahr eine Hyperthermie entwickeln, wenn sie sich im Schatten aufhält. Hält sie sich stattdessen in der Sonne auf, erhöht sich dieser Wert von 4 Tagen auf 36 Tage pro Jahr, wenn sie sich moderat bewegt von 13 auf 58 Tage und schließlich bei körperlich anstrengender Arbeit von 31 auf 94 Tage pro Jahr. Eine derartige Entwicklung führe zu einem Rückgang körperlicher Aktivität auf Populationsebene [17].

Wie uneindeutig und widersprüchlich die Datenlage zur künftigen Gesamtwirkung der Klimaerwärmung ist, zeigt ein weiterer systematischer Review internationaler Studien. Nach Sichtung der weltweiten Studienlage sei im Zuge des Klimawandels insgesamt zumindest bis „zur Mitte oder zum Ende des 21. Jahrhunderts“ [19] von einer Zunahme körperlicher Aktivität in der Freizeit und zum aktiven Transport auszugehen. Dabei bleiben die Autor*innen bei ihrem Fazit bewusst vorsichtig und vermeiden es, den Zeitpunkt konkret zu beziffern, zu dem der zunächst bewegungsförderliche Effekt zunehmender Durchschnittstemperaturen ins Gegenteil umschlagen soll [19]. Erwähnenswert ist schließlich eine Projektionsstudie aus den USA. Diese kommt zu dem Ergebnis, dass die körperliche Freizeitaktivität in den USA netto zumindest über die nächsten 10 Jahre zunehmen werde [7].

## Veränderung körperlicher Aktivität nach Bewegungsdomänen

Zunächst einmal ist festzuhalten, dass die bisher berichteten Zusammenhänge zwischen verschiedenen Klima- und Wetterelementen einerseits und Indikatoren der Gesamtaktivität andererseits im Wesentlichen auch für spezifische Bewegungsdomänen gefunden wurden [18]. Innerhalb und zwischen den Bewegungsdomänen kann sich eine spezifische Wetterlage allerdings unterschiedlich stark auswirken: Dies zeigt sich beispielsweise anschaulich innerhalb der Domäne „aktiver Transport“ beim Vergleich von Radfahrer*innen mit Fußgänger*innen.

In Beobachtungsstudien reagierten Radfahrer*innen auf Regen und vor allem auf Regenschauer deutlich sensibler als Fußgänger*innen. Konkret reduzierten Erstere eher die Zahl und die Distanzen ihrer Fahrten als Letztere [19]. Dies erscheint nicht verwunderlich, da Fußgänger*innen durch Kleidung, Regenschirme und eine geringere Geschwindigkeit gegenüber Regen, Schnee und Wind weniger stark exponiert sind. Gemäß einer kanadischen Studie reagieren im Übrigen vor allem regelmäßige Fußgänger*innen am wenigsten empfindlich auf diesbezügliche Wetterschwankungen [14].

Auch zwischen den Bewegungsdomänen werden unterschiedlich starke Reaktionen auf klimatische Veränderungen diskutiert: Bewegung zum Zwecke des aktiven Transports basiere häufig auf extrinsischen lebensweltlichen Notwendigkeiten (etwa zu Fuß zum Einkaufen zu gehen oder mit dem Fahrrad zur Schule oder Arbeit zu gelangen), während Bewegung in der Freizeit, also zur Erholung oder beim Sport, stärker intrinsisch motiviert sei und deswegen durch die situativen Wetterbedingungen stärker beeinflussbar sein dürfte [25].

## Kontextuelle Faktoren

**Abb. 1:**
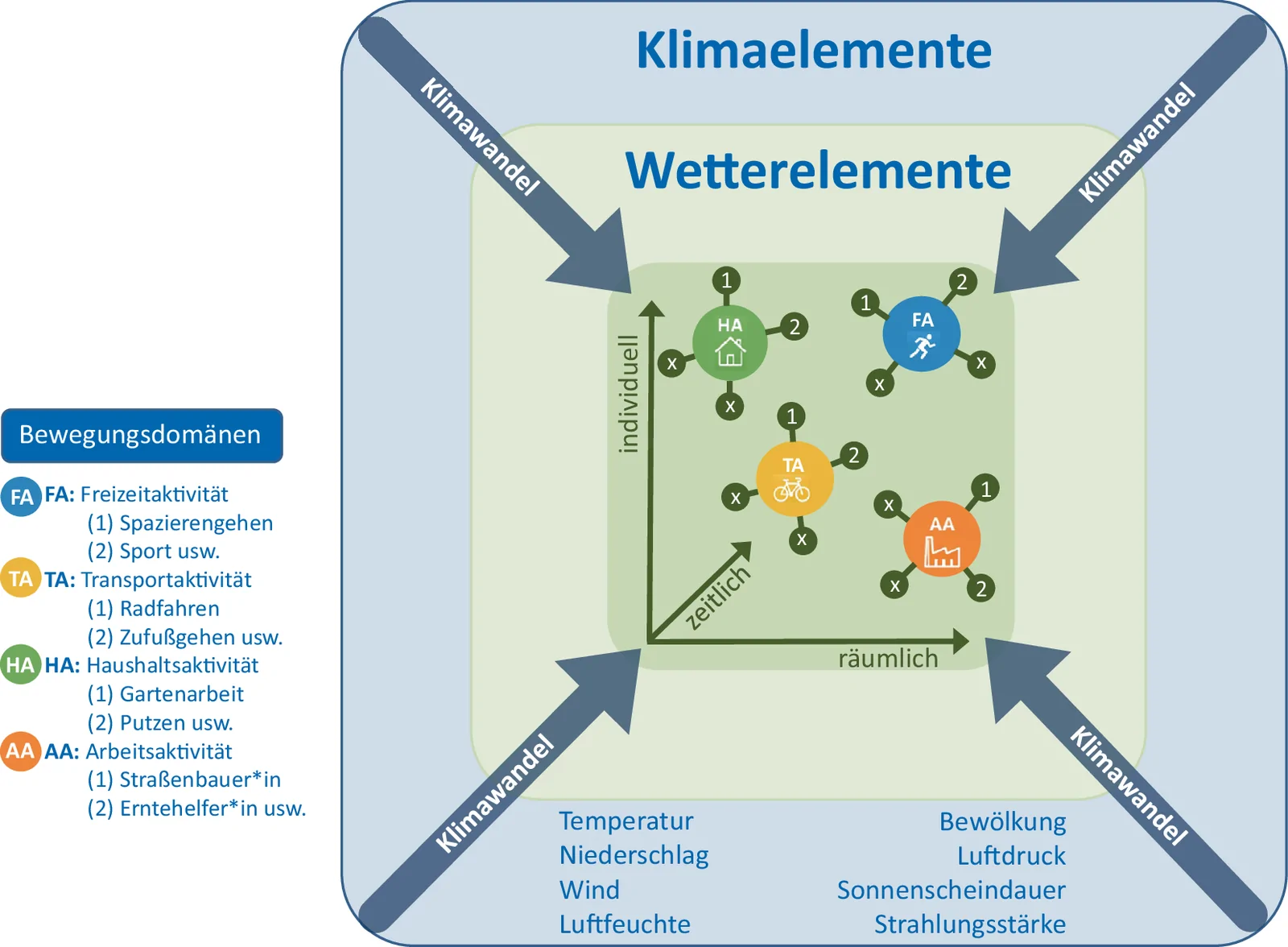
Modell zum Einfluss des Klimawandels auf verschiedene Bewegungsdomänen in ihrer individuellen, zeitlichen und räumlichen Ausprägung

Die Bedeutung des Klimawandels und sein Einfluss auf die mess- und beobachtbaren Klima- und Wetterelemente werden in Abb. 1 veranschaulicht. Sie soll zeigen, dass letztlich das konkrete lokale Wetter sowohl die körperliche Aktivität insgesamt als auch spezifisch innerhalb der einzelnen Bewegungsdomänen beeinflusst. Das konzeptionelle Modell berücksichtigt zudem, dass sich Bewegung (etwa als Zufußgehen, Radfahren, Sport oder körperliche Arbeit) entlang individueller, zeitlicher und räumlicher Dimensionen manifestiert (Abb. 1). Dies spiegelt einen weiteren zentralen Befund aus der vorliegenden Literatur zum künftigen Einfluss des Klimawandels auf körperliche Bewegung wider. So betonen zahlreiche Autor*innen über meteorologische Faktoren hinaus die Bedeutung kontextueller Faktoren auf individueller, zeitlicher und räumlicher Ebene [7, 19, 25]. Die Bedeutung dieser Faktoren veranschaulichen die folgenden 3 Abschnitte.

### Individuelle Faktoren.

Individuelle Unterschiede bestehen beispielsweise hinsichtlich der Vulnerabilität gegenüber Einflüssen des Klimawandels, hinsichtlich des aktuellen Bewegungsverhaltens sowie hinsichtlich der Reaktion auf Klima- und Wetterveränderungen. Laut Weltgesundheitsorganisation zählen Kinder, ältere Menschen, multimorbide Personen ebenso wie Sportler*innen und Außenbeschäftigte zu den besonders vulnerablen Bevölkerungsgruppen hinsichtlich der oben skizzierten klimaassoziierten Gesundheitsrisiken. Als besonders gefährdet gelten darüber hinaus Menschen mit eingeschränkter Mobilität oder Behinderungen, obdachlose Personen sowie Bewohner*innen von schlecht isolierten Wohnungen mit fehlendem Zugang zu schattigen Orten oder zu kühlen Rückzugsräumen [11, 12].

Große Teile dieser Personengruppen sind nicht nur besonders vulnerabel gegenüber Hitze und anderen Wetterelementen, sondern weisen bereits jetzt Defizite in ihrem Bewegungsverhalten auf. So ist bekannt, dass etwa ältere Menschen, sozioökonomisch benachteiligte Personen und Menschen mit chronischen (v. a. metabolischen) Erkrankungen am wenigsten körperlich und sportlich aktiv sind [6].

Diese Ungleichheiten laufen Gefahr, durch den Klimawandel weiter verstärkt zu werden. Projektionen aus den USA und Daten aus Kanada zeigen, dass bei Hitzewellen (dort ab einer Außentemperatur von 36 °C) insbesondere ältere und übergewichtige Personen körperliche Aktivitäten deutlich einschränken [7, 18]. Auch durch respiratorische und kardiovaskuläre Erkrankungen vorbelastete Menschen reagieren besonders sensibel auf Hitze und Luftschadstoffe, indem sie ihre Aktivitäten vom Freien ins Haus verlagern [11, 26].

Dazu passen Daten aus Deutschland, wonach 8 von 10 Personen über 65 Jahren bei einer Hitzewelle ihre körperliche Aktivität als bewusste Anpassungsmaßnahme deutlich reduzieren [27]. Präventionsansätze wie Hitzeaktionspläne als strategische Konzepte für Regionen oder Kommunen oder Musterhitzeschutzpläne als praxisnahe Konzepte für Institutionen sollten also durch spezifische Verhaltensempfehlungen und strukturelle Maßnahmen darauf abzielen, dass sich das vorbestehende Bewegungsdefizit dieser Risikogruppen insbesondere im Sommer und bei Hitzewellen nicht noch weiter verschärft.

### Zeitliche Faktoren.

Beim Blick auf jahreszeitliche Muster ist es nicht verwunderlich, dass das Ausmaß körperlicher Aktivität und sportlicher Betätigung grundsätzlich im Sommer ausgeprägter ist als im Winter [20]. Ein möglicher Anstieg der körperlichen Aktivität infolge künftig milderer Witterung dürfte deswegen vor allem auf einer Zunahme der Aktivität im Winter zurückzuführen sein. Zu diesem Schluss kommt beispielsweise ein systematischer Review, wonach im Sommer u. a. wegen häufigerer Hitzewellen und anderer Extremwetterereignisse körperliche Aktivität insgesamt eher reduziert werde, wohingegen die mildere Witterung in den kälteren Monaten körperliche Aktivität begünstige [19].

### Räumliche Faktoren.

Das Bewegungsverhalten wird darüber hinaus von regionalen und lokalen Faktoren beeinflusst. Die Meteorologie unterscheidet hierzu die oben bereits eingeführten Klimaelemente von sogenannten Klimafaktoren. Zu den Klimafaktoren zählen die geografische Breite, die topografische Höhe und lokale Gegebenheiten wie die Entfernung zu natürlichen oder künstlichen Wasserflächen, die lokale Bebauung sowie Bodenart und -bedeckung. Klima- und Wetterelemente werden von diesen Klimafaktoren beeinflusst, was zu regional und lokal höchst unterschiedlichen mikro- und mesoklimatischen Verhältnissen führt [8, 28].

Auf derartige Unterschiede weist beispielsweise die bereits zitierte Projektionsstudie für die USA hin. Demnach werde in den nördlichen Bundesstaaten die körperliche Aktivität insgesamt aufgrund milderer Temperaturen deutlich zunehmen, dagegen in den heißen Südstaaten insgesamt eher abnehmen [7]. Auch innerhalb Deutschlands variieren die klimatischen Bedingungen regional deutlich. Für den Nordosten unseres Landes sind stärkere Temperaturextreme, kältere Winter, mehr Wind und ausgeprägtere Dürreperioden charakteristisch, während im Südwesten eher ein milderes, feuchteres und windarmes Klima vorherrscht [29].

Lokal wird Bewegung nicht zuletzt von der gebauten Umwelt beeinflusst. Die besonderen Temperaturbedingungen in Städten sind vor allem im Sommer durch den städtischen Wärmeinseleffekt gekennzeichnet [30]. Typisch für den Stadtraum sind sogenannte innerstädtische Wärmeinseln („urban heat islands“), welche sich durch die Aufheizung von Ziegeln, Glas, Beton, Stahl sowie asphaltierten und anderweitig versiegelten Flächen im Verein mit einer geringeren Belüftungseffektivität bilden. Wie stark der städtische Wärmeinseleffekt ist, hängt vor allem von der Größe der Stadt, ihrer Dichte, der Höhe der Bebauung, dem Versiegelungsgrad und dem Grünflächenanteil sowie den verwendeten Baumaterialien (Glas, Beton usw.) ab.

Im Mittel liegen hierzulande die Temperaturen in der Stadt um etwa 0,5 °C bis 2 °C höher als auf dem Land [31]. In Großstädten wie Berlin liegen die täglichen Temperaturunterschiede zwischen Zentrum und Umland im Sommer noch höher: Das Umweltbundesamt hat hier schon Temperaturdifferenzen von bis zu +11 °C dokumentiert [31]. Inwiefern sich Wärmeinseleffekte hierzulande durch das Zusammenwirkung von Klimawandel und fortschreitender Urbanisierung verstärken werden, ist nach Einschätzung des Deutschen Wetterdienstes noch offen [30, 31].

## Fazit und Ausblick

Schon heute wird aus Public-Health-Sicht das gesundheitsförderliche Potenzial körperlicher Bewegung nicht ausreichend genutzt. Der Temperaturanstieg und weitere klimatische Veränderungen drohen, bestehende Bewegungsdefizite weiter zu verstärken.

Eine zentrale Rolle spielt in diesem Zusammenhang die künftige Veränderung der Lufttemperatur. Während steigende Außentemperaturen Bewegung bis zu einem gewissen Punkt begünstigen, scheinen heiße Tage den gegenteiligen Effekt zu haben. So legen bisherige Studien nahe, dass körperliche Aktivität bei akuten Hitzewellen deutlich eingeschränkt wird. Der typische umgekehrt u‑förmige Zusammenhang zwischen Lufttemperatur und Bewegung wird dabei durch weitere Klimaelemente ebenso beeinflusst wie durch individuelle, zeitliche und regionale Faktoren, was eine Prognose des künftigen Bewegungsverhaltens erschwert. So verwundert es nicht, dass sich Bewegung und körperliche Aktivität zwar retrospektiv gut modellieren lassen, Projektionen aber zu unterschiedlichen, teils widersprüchlichen Ergebnissen kommen [15]. Somit bleibt Stand heute offen, in welchem Zeitfenster und in welchem Setting insgesamt eher von einer Zu- oder von einer Abnahme körperlicher Aktivität – etwa in Deutschland – auszugehen ist. Das abschließend entwickelte konzeptionelle Modell soll die Differenzierung, Operationalisierung und Systematisierung künftiger Forschungsarbeiten zu dieser Thematik unterstützen.

## Data Availability

Für diesen Artikel wurden keine Datensätze erstellt oder analysiert.
